# Non-coding RNA in *C9orf72*-related amyotrophic lateral sclerosis and frontotemporal dementia: A perfect storm of dysfunction

**DOI:** 10.1016/j.ncrna.2018.09.001

**Published:** 2018-09-10

**Authors:** Andrew G.L. Douglas

**Affiliations:** a Wessex Clinical Genetics Service, University Hospital Southampton NHS Foundation Trust, Southampton, UK; b Human Development and Health, Faculty of Medicine, University of Southampton, Southampton, UK

**Keywords:** C9orf72, Amyotrophic lateral sclerosis, ALS, Frontotemporal dementia, FTD, Non-coding RNA

## Abstract

A hexanucleotide repeat expansion in the first intron/promoter region of *C9orf72* is the most common genetic cause of amyotrophic lateral sclerosis (ALS) and frontotemporal dementia (FTD). Both sense and antisense transcripts exist at the *C9orf72* locus but the function of the antisense lncRNA is unknown. RNA toxicity of the transcribed repeat expansion has been implicated in the pathogenesis of *C9orf72*-related ALS/FTD, not only through direct sequestration of important RNA binding proteins but also indirectly through non-ATG dependent translation into dipeptide repeats. Formation of RNA/DNA hybrid R-loops may also play a key role in the pathogenesis of this condition and this mechanism could provide a link between the repeat expansion, DNA damage, repeat instability and deficiency of RNA binding proteins. Non-coding *C9orf72* antisense transcripts could also act to epigenetically regulate gene expression at the locus. The potential effects of such non-coding RNAs should be considered in the design of antisense oligonucleotide therapeutics for *C9orf72*-related ALS/FTD. Furthermore, the mechanisms of RNA dysregulation exemplified by *C9orf72*-related disease may help illustrate more broadly how a “perfect storm” of dysfunction occurs in ALS/FTD and how targeting these factors could lead to corrective or preventative therapies.

## 1 Introduction

Amyotrophic lateral sclerosis (ALS), also known as motor neurone disease (MND), is a progressive neurodegenerative disease of motor neurones. It is typically a disease of late adulthood, with onset peaking between 50 and 75 years of age, and in most cases it has a rapidly progressive course with average survival after diagnosis of only around 3–5 years [[1], [2], [3]]. Around 5% of ALS cases appear to be familial, exhibiting an autosomal dominant pattern of inheritance [4,5]. There is, however, a significant degree of incomplete penetrance among such families [6]. While this in itself is not unusual for an inherited late-onset condition, the issue of penetrance is further compounded by the phenotypic spectrum of conditions associated with inherited ALS-causing mutations. In particular, some 5–15% of ALS patients also receive a diagnosis of frontotemporal dementia (FTD) and up to 50% of patients experience FTD-like symptoms of some kind [7]. FTD is the third most common type of dementia after Alzheimer disease and Lewy body dementia [8]. Frontal and temporal lobe atrophy leads to a cognitive-behavioural phenotype of disinhibition, apathy, personality change and language disturbance. Around 12.5% of FTD patients also have ALS and up to 40% have at least some features of the condition [9]. Similar to ALS, at least 10% of FTD cases exhibit autosomal dominant inheritance and up to 40% of cases exhibit some degree of family history. It is now recognised that ALS and FTD are at two ends of a disease spectrum, with mutations in many of the same shared genes giving rise to both conditions and with variable presentations arising, even within a single family [10].

Despite the clinical phenotypes of ALS and FTD being markedly different from each other, both conditions share key features at the pathological level, including in most ALS cases and in many FTD cases a characteristic cellular pattern of TDP-43 proteinopathy [11,12]. In addition, evidence points in both cases towards a role for the pathological spread of disease through the central nervous system (CNS) [13]. In ALS, this propagation of pathology has been shown to occur, in a somewhat prion-like manner, by both contiguous cell-to-cell spread and by network spread along synaptic pathways, with such spread corresponding to the progression from focal to more generalised clinical signs and symptoms [14]. In FTD, there is also evidence of similar pathological spread within the brain [15,16]. Thus, the predominant clinical presentation of any given patient within the ALS/FTD spectrum is likely to reflect the initial brain or spinal cord region affected, with clinical progression being linked to the subsequent pathological spread of the required conditions for neurodegeneration within the CNS.

Over 25 different genes have so far been identified in relation to familial ALS/FTD [6,17,18]. The most significant of these to date has been the identification of a hexanucleotide (GGGGCC)_n_ expansion (>30 repeats being classed as pathogenic) within the first intronic region of the *C9orf72* gene [19,20]. The discovery that this expansion is found in up to 40% of familial ALS cases and up to 25% of familial FTD cases makes it by far the biggest single genetic cause of ALS/FTD [21]. Furthermore, the *C9orf72* expansion is also identified in up to around 6% of apparently sporadic ALS cases and 6% of sporadic FTD. This unexpected link to sporadic disease is believed to be partially due to incomplete or inadequate family history information being available and also because of reduced penetrance owing to the late onset of ALS and FTD. However, population studies have suggested that up to 0.2–0.6% of the North European population may in fact carry the *C9orf72* expansion, a carrier frequency far in excess of what would be expected and one similar to the overall lifetime risk of developing ALS [22,23]. Since the discovery of *C9orf72* in 2011, it has therefore gradually become clear that significant variability of penetrance exists for the phenotypes associated with this mutation and that modifying mutations and variants in other ALS/FTD-related genes are often additionally present in affected expansion-positive patients [6].

The underlying pathogenetic mechanism of the *C9orf72* expansion (and indeed that of all ALS/FTD) remains the subject of intense ongoing research across the globe. However, a number of common themes have emerged in relation to neurodegenerative diseases in general and to ALS/FTD in particular. Principal among these is the seemingly central role of RNA in disease, whether it be abnormal pre-mRNA splicing, abnormal RNA transport, microRNA dysregulation, repeat-associated non-ATG-dependent (RAN) translation or the sequestration of important cellular factors by toxic non-coding RNA. In this review, we shall examine the various roles of RNA in *C9orf72*-related ALS/FTD and consider what this may mean for the understanding of this devastating neurodegenerative disorder and what implications there may be for the design of targeted therapeutics.

## 2 *C9orf72* in its genomic context

The function of the C9orf72 protein has yet to be fully elucidated. Bioinformatic and experimental evidence supports a role for it in vesicular trafficking as a Rab-GTPase exchange factor (Rab-GEF) and indeed an interaction with Rab proteins has been demonstrated, as well as the protein showing a regulatory effect on autophagy and extracellular vesicle release [[24], [25], [26], [27]]. C9orf72 protein has also been shown to be necessary for the formation of stress granules, suggesting that reduced expression could impact upon stress response [28]. The gene has orthologues in organisms as divergent as *C. elegans*, suggesting a key conserved role in multicellular animals, although no such orthologue appears to exist in *Drosophila melanogaster* [25,29]. Interestingly, mice that are homozygous knockouts for their orthologue of *C9orf72* (*3110043O21Rik*) do not in fact exhibit a neurological phenotype but instead develop an unusual autoimmune lymphoproliferative disorder and macrophage/microglial dysfunction [[30], [31], [32]]. Similarly, no loss-of-function mutations in *C9orf72* have been identified in any ALS/FTD patient cohorts to date [33]. Thus, the current consensus is that while *C9orf72* loss of function may play an important role in the disease, and although a decrease in gene expression has been seen in the brain in the presence of the expansion, the loss-of-function mechanism on its own is not the sole pathogenic driver of the condition [20,34].

The GC-rich nature of the *C9orf72* repeat and its similarity to the (CGG)_n_ repeat found in fragile X syndrome led to the suspicion that large expansions might induce DNA hypermethylation at the gene's locus. Indeed, it has been known for many years that a rare folate-sensitive chromosome fragile site exists at the 9p21 locus, the same genomic region as the *C9orf72* locus [35,36]. This particular fragile site has up until now never been molecularly characterised and may well therefore represent the presence of the *C9orf72* expansion itself. Hypermethylation of a CpG island upstream of the expansion was indeed confirmed [37]. However, a larger CpG island exists downstream of the expansion and this has not been shown to be hypermethylated. The purpose and significance of this second CpG island remains unknown. The repeat expansion itself is of course another CpG-rich region. However, it appears that the repeat's CpGs themselves are not hypermethylated [38].

Histone modifications have also been demonstrated in *C9orf72* expansion-positive cases, with repressive histone trimethylation marks H3K9me3, H3K27me3, H3K79me3 and H4K20me3 being shown to be enriched in the brains of affected patients [39,40]. Interestingly, there was much stronger correlation between presence of these repressive chromatin marks and reduced *C9orf72* expression than the corresponding levels of DNA methylation, suggesting that chromatin confirmation rather than DNA methylation *per se* is more immediately relevant in determining the gene's expression.

## 3 Coding and non-coding transcripts of *C9orf72*

**Fig. 1:**
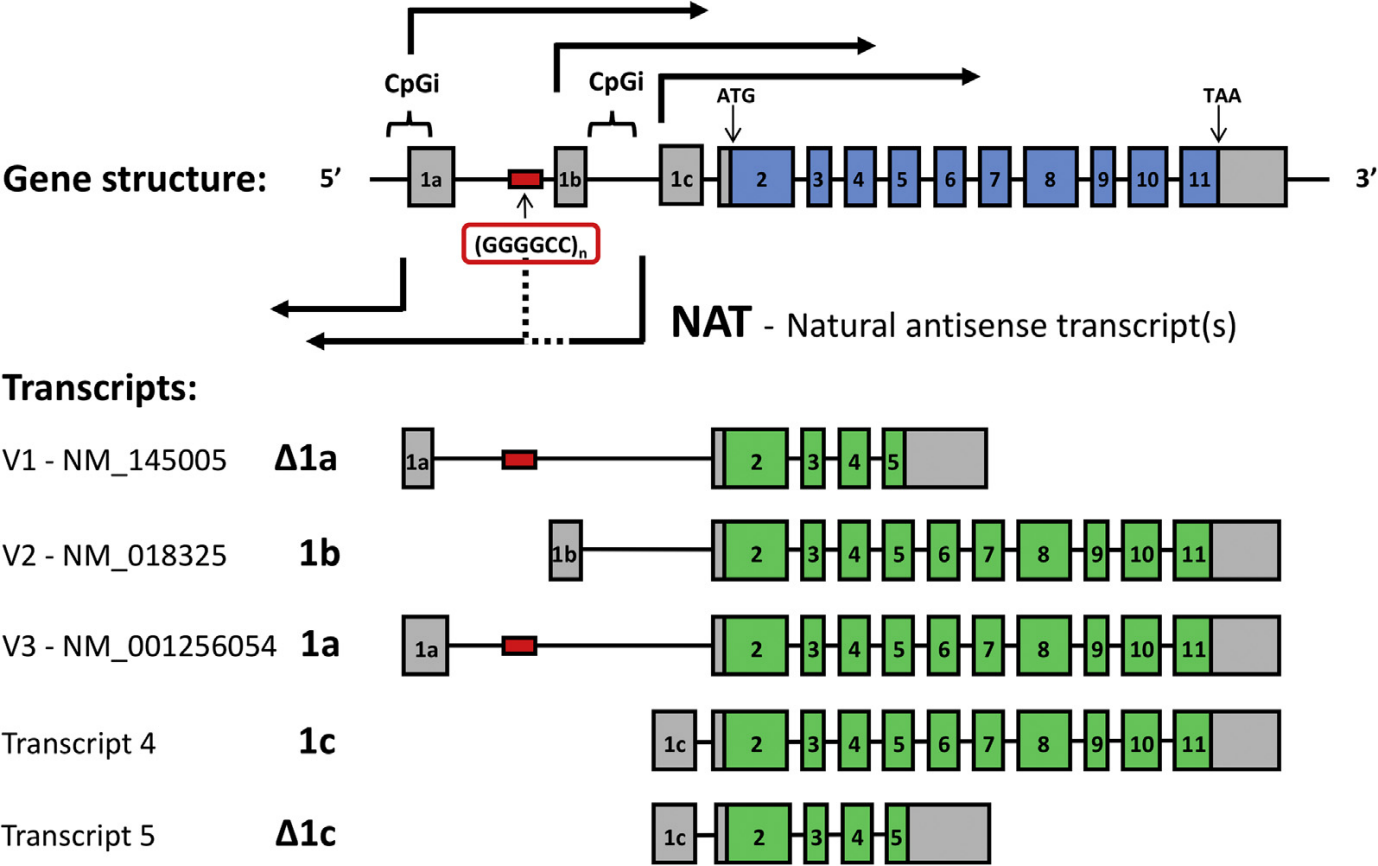
**Transcripts of *C9orf72***. **A**. Human *C9orf7*2 has three annotated RefSeq transcripts but at least two additional transcripts utilising an alternative first exon 1c have also been described (transcripts 4 and 5), as have natural antisense transcripts (NATs) [46].

At the time of the initial reports of the link between *C9orf72* and ALS/FTD, three annotated transcripts of the gene had been described (Fig. 1) [19,20]. V1 (NM_145005) starts upstream of the hexanucleotide repeat and comprises a shortened first exon (referred to here as Δ1a), subsequently terminating with an extended exon 5 which, by not utilising the intron 5 splice donor site, results in the incorporation of a premature stop codon immediately downstream of the missed splice site. V2 (NM_018325) is the predominantly expressed transcript of *C9orf72* and comprises the full 11 exons of the gene but starts with an alternative exon 1b that lies downstream of the hexanucleotide repeat. V3 (NM_001256054) again incorporates 11 exons but utilises the full-length exon 1a by using an alternative intron 1 splice donor site downstream of that used for Δ1a.

A notable observation with regards *C9orf72* expression is that transcript V2, which starts downstream of the expansion, accounts for the vast majority (92.6%) of all *C9orf72* transcripts as determined by ENCODE CAGE-seq data [38]. Some studies have found levels of V1 to be very low compared to V2 using qRT-PCR and ddPCR methods [39]. However, different studies have reported similar levels of V1 and V2 using Nanostring counting technology [41]. Yet other studies have reported a shift to preferential usage of exon 1a over exon 1b in the presence of the repeat expansion [42]. The cause for this discrepancy in reported isoform abundances remains unclear. However, it may partly reflect a limitation in PCR-based mRNA quantification methods that rely on correct transcript splicing, since intron retention of the repeat-containing intron 1 is now known to occur in the presence of the expansion [43]. Similar findings of intron retention have been seen in myotonic dystrophy type 2 and the prospect of using such splicing features as disease biomarkers has been raised [44]. An intriguing link can also be made to TDP-43, which is the hallmark protein associated with ALS pathology [12]. Mislocalisation of TDP-43 from the nucleus to the cytoplasm is seen in the majority of patients with ALS, including *C9orf72* cases, and it is known that TDP-43 loss of function tends to lead to the retention of long introns [45].

**Fig. 2:**
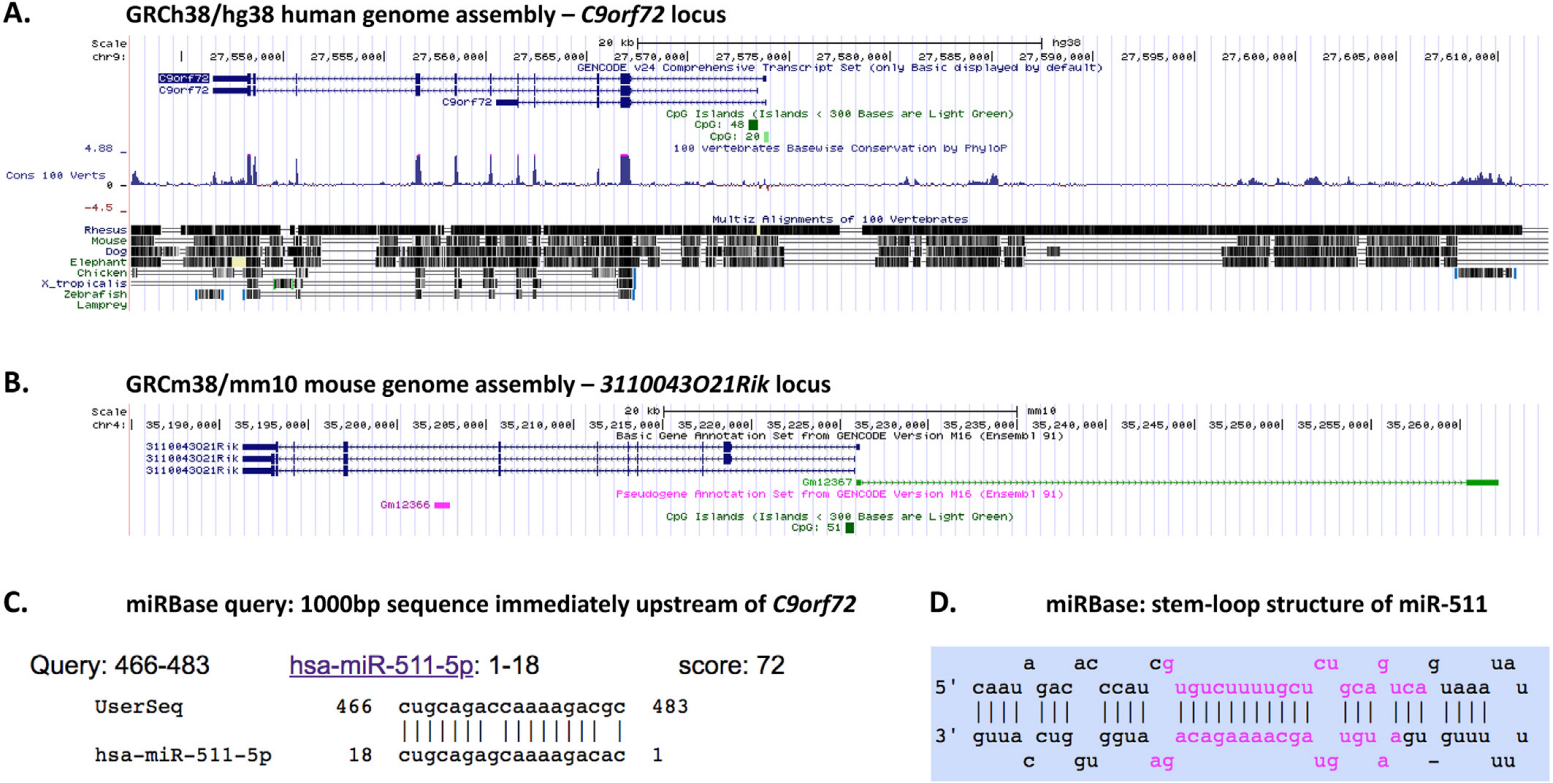
**The region upstream of the *C9orf72* locus**. **A**. A degree of sequence conservation is present among mammals upstream of *C9orf72*. **B**. The mouse *C9orf72* orthologue locus (*3110043O21Rik*) also exhibits a NAT (*Gm12367*). **C**. miRBase analysis of the kilobase of sequence immediately upstream of *C9orf72* exon 1a reveals a putative miR-511-5p binding site. **D**. The stem-loop structure of miR-511 (image from miRbase).

In addition to the three commonly described transcripts, at least two other isoforms have been identified that use another alternatively spliced first exon (exon 1c) that lies downstream of exon 1b [46]. Furthermore, one or more antisense transcripts have been described that arise from the same promoter region as the sense transcripts but where transcription proceeds in the opposite direction. Indeed, in the presence of the hexanucleotide expansion, both sense (GGGGCC)_n_ and antisense (CCCCGG)_n_ RNA foci are seen within cell nuclei, confirming that antisense transcription takes place [41,42,[47], [48], [49], [50]]. The function of the *C9orf72* antisense lncRNA is unknown. However, a similar antisense transcript has been found in mice (*Gm12367*) and analysis of comparative genomic alignments points towards a degree of conservation of the region immediately upstream of *C9orf72* among eutherian mammals (Fig. 2).

It has recently been recognised that conserved sequences within lncRNAs can in some instances represent miRNA binding sites [51]. Furthermore, such sites can in fact act to downregulate the target-binding miRNA, rather than the more traditional and opposite effect of the miRNA downregulating its target. Examination of the first kilobase of sequence immediately upstream of the first exon of *C9orf72* using the search function of the miRBase database of miRNAs (www.mirbase.org) reveals a putative miR-511-5p binding site 518–535 nucleotides 5’ to the exon 1a start site (Fig. 2) [52]. Although this sequence contains a mismatch of the second seed-region nucleotide sequence of miR-511-5p, the remaining complementarity of the sequence may be convincing enough to warrant further study, especially as mir-511 is known to be downregulated in conditions such as Alzheimer disease, has a regulatory role in neuronal differentiation and appears important in regulating monocyte/macrophage inflammatory responses [[53], [54], [55], [56]].

## 4 RNA toxicity

Cellular toxicity of transcribed repeat expansions is an increasingly recognised disease mechanism, particularly among neurogenetic conditions such as myotonic dystrophy [57]. Repetitive RNA sequences adopt different secondary and tertiary structures depending on their sequence and these molecules form RNA foci within cell nuclei [57]. Here they can bind and sequester important cellular factors, leading to secondary dysregulation of processes such as transcription, splicing and RNA transport. RNA foci are invariably reported in the presence of the *C9orf72* repeat expansion and it appears that each focus represents a single molecule of the transcribed expansion [19,20,58]. The (GGGGCC)_n_ sequence in *C9orf72* lends itself to formation of RNA G-quadruplexes and such structures have been identified *in vitro* using such sequences [[59], [60], [61]]. G-quadruplexes can take one of several conformations and in its DNA form the *C9orf72* repeat has been shown to adopt two distinct antiparallel quadruplexes both with four G-quartets and three lateral (edgewise) loops, while the RNA version of the repeat has been shown to adopt a parallel quadruplex topology again with stacks of four G-quartets but this time linked with three propeller (chain-reversal) loops [62]. It is known that the antisense sequence of the repeat (CCCCGG)_n_ is transcribed and forms antisense foci but these antisense RNA repeats cannot form G-quadruplexes and their structural conformation has not yet been formally resolved [50]. While the C-rich antisense DNA strand of the repeat has been shown to form an i-motif structure, RNA is thought less likely to form such structures under physiological conditions [[63], [64], [65]].

Despite multiple attempts, it has proved somewhat difficult to consistently identify the factors binding to RNA foci in *C9orf72* expansion-positive cells. This is likely to partially reflect the relative promiscuity with which RNA-binding proteins (RBPs) bind to RNA. Reported binding factors include ADARBP, hnRNP-H, SRSF1, hnRNP-A3, hnRNP-A1, Pur-α, SRSF2, hnRNP-F, ALYREF and nucleolin [41,42,60,[66], [67], [68], [69], [70], [71]]. Among the factors that have been reported to bind by multiple independent studies, one appears to be Pur-α, an ubiquitously expressed multifunctional protein that among other things acts as a transcriptional regulator. Pur-α also interacts with SRSF1, a splicing factor that has itself been implicated in binding *C9orf7*2 RNA foci. Another particular RBP that has been identified by multiple studies to bind the *C9orf72* repeat expansion is heterogeneous ribonucleoprotein H (hnRNP-H) [66,[72], [73], [74]]. hnRNP-H sequestration has been shown to lead to abnormal splicing of its known targets within the brains of *C9orf72* patients [74]. Furthermore, the magnitude of splicing dysregulation in these patients has been shown to broadly correlate with disease severity, raising the prospect that analysis of splicing could provide a quantitative measure or indeed a biomarker for ALS/FTD.

If sequestration and cellular depletion of hnRNP-H is indeed a key factor in ALS/FTD pathogenesis, how could this effect be mediated? Looking at the known targets of hnRNP-H, one intriguing possibility may relate to alternative splicing of telomere repeat-binding factor 2 (TRF2) [75]. TRF2 is a telomere-stabilising protein, acting as a scaffold and hub for recruitment of multiple proteins including a complex of shelterin proteins that protect telomeres. While the full-length TRF2 protein binds telomeric DNA within the nucleus, an alternatively spliced C-terminal truncated isoform (TRF2-S) is expressed in cytoplasm during neuronal differentiation [76]. TRF2-S preferentially binds RNA and is present within axons where it regulates mRNA trafficking. The switch from TRF2 to TRF2-S depends on use of an alternative 5’ splice site within exon 7 that results in a premature termination codon in exon 8. TRF2 is preferentially produced in the presence of hnRNP-H, while in its relative absence there is a shift towards TRF2-S [77]. Thus, in *C9orf72* expansion-positive neurones, where RNA foci sequester hnRNP-H, one might expect increased levels of TRF2-S during neuronal development, which might in turn lead to dysregulation of mRNA transport, a mechanism that has been implicated previously in ALS pathogenesis. Similarly, should a corresponding reduction in TRF2 occur, this might lead to activation of the DNA damage response due to telomere instability, with the possibility of premature neuronal senescence.

In addition to direct RNA toxicity, *C9orf72* repeats have been shown to undergo RAN translation leading to the aggregation of dipeptide repeats, which themselves have toxic properties and have been linked to a neurodegenerative eye phenotype in fruit fly models [50,[78], [79], [80]]. Arginine-containing dipeptide repeats such as poly-GR and poly-PR appear to be especially toxic and they have been reported to interfere with the dynamics of membrane-lacking organelles such as stress granules and nucleoli [[81], [82], [83]]. The role of dipeptide repeats in ALS/FTD pathogenesis remains to be fully clarified. However, nucleolar stress, translation inhibition, rRNA suppression, spliceosome and stress granule abnormalities, dysfunctional nucleocytoplasmic transport, abnormal Notch signalling and dysfunction of the ubiquitin-proteasome system have all been implicated [81,[84], [85], [86], [87], [88], [89], [90]].

## 5 R-loop formation

There is a propensity for expanded repeats within DNA to form so-called “R-loop” structures during transcription [91]. This is where transcribed RNA across a repeat region remains hybridised to its complementary DNA strand. This forms a bulge in the DNA since the dsDNA helix cannot re-anneal. Concurrently, the previously transcribed RNA upstream of the repeat disassociates from its template and this tail along with the aforementioned bulge adopts something akin to an “R”-shaped loop. Naturally-occurring R-loops are found throughout the human genome and play an important cellular role in directing chromatin modifications, replication of mitochondrial DNA and termination of transcription [[92], [93], [94]]. However, in the setting of a long repeat expansion, stable R-loops may preferentially form. This impedes the subsequent progress of transcriptional machinery at the locus, which thereby decreases gene expression. Since both sense and antisense transcription occur at the *C9orf72* repeat locus, such R-loops can in principle form in either or in both directions. Indeed, R-loop formation has even been shown to promote antisense transcription itself [95].

Notwithstanding the potential mechanism linking *C9orf72* and hnRNP-H discussed previously, there is likely to be a more generalisable connection between RBP dysregulation and R-loop formation in ALS/FTD; one which concerns genome integrity and DNA repair [96]. Aside from directly interfering in transcription, R-loops also predispose to DNA damage and trigger repeat instability [97]. Such damage has been reported to occur not only because of DNA replication forks stalling on encountering R-loops within replicating cells, but also through direct aberrant engagement of DNA repair factors by transcription-induced secondary structures, which might occur in non-dividing cells such as neurones [[98], [99], [100]]. Stalling of DNA replication forks can lead to double-strand DNA breaks, while formation of RNA-DNA hybrids leaves the remaining unpaired DNA strand vulnerable to damage. Furthermore, the binding of nuclear RBPs to nascently transcribed RNA appears to help prevent R-loop formation. The splicing factor SRSF1, which is among those RBPs thought to be sequestered by *C9orf7*2 RNA foci, appears to help prevent R-loop formation in this way and it has been shown that depleted SRSF1 levels are associated with increased numbers of R-loops [101].

Intriguingly, a number of other ALS-predisposing genes also have links to either R-loop biology or to DNA repair processes. Senataxin, encoded by *SETX* and a known cause of juvenile-onset ALS, is an RNA helicase that actively resolves R-loop structures [102,103]. It has also been suggested that *ATXN2*, whose polyglutamine repeat expansion can be a modifying factor in ALS, might be involved in R-loop resolution in a manner akin to its yeast orthologue *Pbp1* [104]. SFPQ, in which intron retention appears to be a hallmark in both familial and sporadic ALS, is a splicing factor found in paraspeckles and interacts with proteins involved in double-strand break repair [[105], [106], [107]]. Matrin 3 (encoded by *MATR3* and a rare cause of ALS) is a target of ATM, an important kinase involved in the repair of double-strand breaks in DNA [108]. Finally, FUS protein, whose mutant form is a well-known cause of familial ALS, also tends to accumulate at double-strand breaks and recruitment of FUS appears to be necessary for proper regulation of histone acetylation and chromatin remodelling during DNA repair [109].

## 6 Non-coding RNA and *C9orf72* epigenetic regulation

While the function of the antisense lncRNA transcribed from the *C9orf72* promoter remains unknown, there are several possibilities that may be considered. For example, a number of such antisense transcripts have been found to play a regulatory role in the expression of their sense counterparts [[110], [111], [112], [113], [114]]. How could such regulation take place? Perhaps the most obvious potential mechanism for this would be where sense and antisense transcripts partially overlap. In this situation, one might expect complementary RNA sequences to hybridise to form dsRNA and such sequences could then activate the RNA interference pathway [115,116]. Secondly, the action of antisense transcription could lead to transcriptional interference of the sense transcript [117]. This could potentially occur via displacement of sense transcription factors by progression of the antisense polymerase through the sense promoter region. Thirdly, it has been shown that some antisense lncRNAs play a role in epigenetic regulation of their corresponding sense transcript [[110], [111], [112]]. These lncRNAs may recruit chromatin-remodelling factors to the shared promoter region of their locus and these factors may in turn modify histones with repressive or activating marks.

The mechanism by which natural antisense transcript (NAT) expression can lead to chromatin modifications has not been fully elucidated. However, lncRNAs are known to be involved in recruiting polycomb repressive complexes 1 and 2 (PRC1 and PRC2) to specific gene loci [118]. PRC2 recruitment leads to di- and trimethylation of H3K27 (note that H3K27me3 is an upregulated repressive chromatin mark in *C9orf72* expansion-positive patients) and PRC1 is involved in chromatin compaction through monoubiquitination of lysine 119 of histone H2A (H2AK119ub). Interestingly, heterochromatin formation has also been linked to R-loop formation, with repressive H3K9me2 marks being formed in HeLa cells at the sites of transcriptional pausing in association with recruitment of components of the RNA interference system [95].

## 7 Targeting *C9orf72* non-coding RNA as a therapeutic strategy

Antisense oligonucleotides (ASOs) are short (generally <30 base-long) synthetic nucleic acid analogues that mimic the molecular structure of DNA and RNA, allowing them to base-pair with complementary native RNA target sequences. ASOs are a highly versatile class of molecules, whose sequence, chemistry and overall design can be engineered to suit multiple different circumstances and to engage multiple different cellular pathways [119,120]. To date, clinical use of ASOs within the setting of neurological disease has led to the development of splice-switching oligonucleotides for the treatment of spinal muscular atrophy (nusinersen) and Duchenne muscular dystrophy (eteplirsen), whose primary action lies in binding and blocking critical splicing elements in their target pre-mRNAs [[121], [122], [123]]. ASOs utilising RNase H-mediated target knockdown have also shown great promise in clinical trials for Huntington disease, where target mRNA is actively degraded [124,125]. In ALS, ASOs targeting knockdown of mutant *SOD1* transcripts have also now commenced clinical trials following favourable first-in-man phase I studies [126].

As soon as the *C9orf72* repeat expansion was identified, multiple groups immediately set about trying to knock down the repeat-containing transcript using ASOs to see whether this could provide a viable therapeutic avenue for an otherwise incurable disease. A number of studies demonstrated that the burden of RNA foci within cells could be reduced by treatment with antisense gapmer-style ASOs targeting the repeat-containing *C9orf72* transcripts [41,42,127]. This approach of course assumes that the foci themselves are the primary cause of the pathology or are at least a helpful proxy readout for the underlying cause. However, if haploinsufficiency were to be playing a pathogenic role then knocking down *C9orf72* transcripts may not adequately treat the condition. Furthermore, it has been reported that higher levels in frontal cortex and cerebellum of transcript V1, whose pre-mRNA will contain the repeat expansion and could therefore potentially be knocked down by repeat-targeting ASOs, is in fact correlated with a survival advantage following onset of disease [128]. This finding is likely to be one reason why clinical trials of *C9orf72*-targeting ASOs have yet to be fully commenced.

Whilst most investigators into *C9orf72* therapeutics have focussed on the direct approach of knocking down (GGGGCC)_n_-containing repeat transcripts, relatively little attention has been paid to alternative ASO approaches. If aberrant R-loop formation turns out to be a key pathogenic factor in *C9orf72*-related ALS/FTD, then ASO chemistries capable of preventing or otherwise abrogating such R-loop formation should be investigated for their therapeutic potential, as has been seen previously in myotonic dystrophy not only with LNA-gapmer but also with LNA-mixmer oligonucleotides [129]. Intriguingly, this study also suggested that somatic repeat instability could be stabilised by administration of sterically blocking ASOs such as LNA-mixmer, phosphorodiamidate morpholino and 2′O-methyl phosphorothioate ASOs, which could prove similarly useful in *C9orf72*. Furthermore, if natural antisense transcript (NAT) expression at the *C9orf72* locus is indeed involved in regulating expression of the gene's sense transcripts, whether by dsRNA formation, transcriptional interference or induction of epigenetic repressive marks, ASOs designed to knock down the antisense transcript may in fact prove to be of therapeutic benefit via normalisation of previously haploinsufficient expression levels.

## 8 Conclusion: a perfect storm of dysfunction

**Fig. 3:**
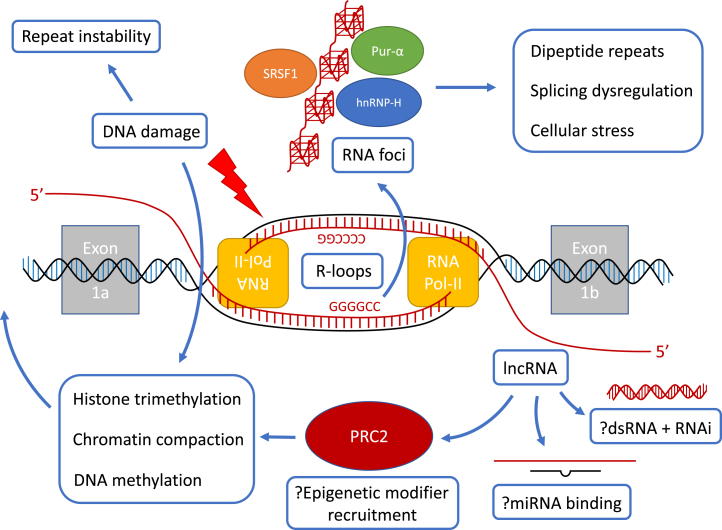
**Potential non-coding RNA effects in *C9orf72*-related ALS/FTD**. Bidirectional transcription across the (GGGGCC)_n_ repeat expansion may lead to aberrant R-loop formation, predisposing the exposed DNA strands to damage, which in turn can lead to repeat instability through aberrant DNA repair. Bidirectional transcription may also lead to transcriptional interference between sense and antisense transcripts. Polymerase stalling may generate abortive transcripts and in the sense direction these can form G-quadruplex-containing RNA foci, which sequester RNA binding proteins such as SRSF1, Pur-α and hnRNP-H, leading to splicing dysregulation. Concurrently, repeat-containing transcripts undergo repeat-associated non-ATG dependent translation, forming dipeptide repeats that impact upon cellular stress at multiple levels such as through impaired nucleocytoplasmic transport and ubiquitin-proteasome function and through nucleolar stress. The natural antisense transcript of *C9orf72* may induce downregulation through the RNAi pathway and may also potentially bind miRNAs relevant to neuronal function. Epigenetic modifiers such as the repressive PRC2 complex may be recruited to the locus, leading to histone trimethylation, chromatin compaction and DNA methylation.

In many ways, the story of *C9orf7*2 has been a case of the gene confounding those studying it at every turn. Firstly, the repeat expansion itself is hard to model owing to its large size, G-C content and inherent instability. Secondly, the disease it causes (ALS/FTD) is only partially understood in terms of its aetiology and therefore it is hard to interpret the contribution of the expansion to disease pathogenesis. Furthermore, the behaviour of the repeat expansion and its clinical effects do not appear to conform to what we have learnt from other repeat expansion disorders, either in terms of genetic anticipation or in relation to a correlation between expansion size and age of onset. However, as more becomes known about the molecular underpinnings of ALS/FTD in all its forms and about *C9orf72* in particular, a fuller and more rounded pathogenetic picture is starting to become clear (Fig. 3). Perhaps most clear of all is that ALS/FTD is not a disease that is caused by any one single factor in isolation. Indeed, it has been shown epidemiologically that some six separate pathogenetic “steps” are required in order to cause ALS [130]. Whilst the exact order and details of this multistep process remain to be determined, it is quite likely that we in fact already know in principle what these steps are. For example, it seems certain that global RNA misprocessing events, secondary to multiple factors such as RBP mutations and/or sequestration, are invariably present in ALS/FTD. Similarly, increased levels of cellular stress, potentially caused via multiple processes such as inefficiencies in the ubiquitin-proteasome system or in autophagy or in stress granule formation, is also a required feature for disease. Other factors such as synaptic dysfunction, inflammation and abnormal glial function could all feed into this mix over time, until a “perfect storm” of dysfunction breaches a pathophysiological threshold that leads to neurodegeneration.

The centrality of RNA misprocessing in ALS/FTD has been highlighted by the finding that altered splicing appears to be a consistent finding across nearly all such cases [105,131,132]. This opens up a potential role for the use of RNA sequencing (RNA-seq) in the diagnosis and monitoring of the disease. By performing transcriptomic analysis of splicing events, it may be possible to detect specific patterns of abnormal splicing that are suggestive of ALS or FTD or even of the involvement of a specific ALS/FTD gene. Since many ALS/FTD genes are involved in fundamental RNA-processing events, it is quite likely that such effects may be detectable in tissues other than the brain and spinal cord, such as blood, which could then potentially provide a clinically useful diagnostic test. In the soon-anticipated era of disease-modifying therapeutics for this condition, a functional readout of RNA processing might also serve as a biomarker indicating response to such therapies.

From a non-coding RNA point of view, then, what key questions should be answered in relation to *C9orf72*? This review helps highlight several potential avenues for further investigation. Firstly, the antisense lncRNA of *C9orf72* should be studied in more detail in order to ascertain its natural function and its potential role in controlling *C9orf72* expression. This might include consideration of potential secondary miRNA effects as well as possible roles in epigenetic regulation. Secondly, studying the role of hnRNP-H and other specific RBPs in relation to *C9orf72* and the effects of their sequestration by the expanded repeat should help elucidate some of the key RNA misprocessing events in this condition. Thirdly, the role of R-loop formation in this disease should be studied in more detail to ascertain its relationship to DNA damage, repeat instability, various RBP deficiencies and the potential for ASO therapeutics.

Finally, it is worth noting that although *C9orf72*-related ALS/FTD is a disease that is hard to study and whose pathogenesis is hard to elucidate, owing to its many layers of complexity, such complexity is in itself also a cause for hope. For just as the perfect storm can be calmed or prevented through dissipation of just one factor, so it may prove that *C9orf72*-related ALS/FTD (or indeed ALS/FTD more generally) will be treatable or preventable by correction of just one of its many pathogenic components.
